# Quality Information for Improved Health

**DOI:** 10.1371/journal.pbio.0020048

**Published:** 2004-02-17

**Authors:** Patricia L Thibodeau, Carla J Funk

## Abstract

The Medical Library Association converts access to information into access to knowledge in a networked environment of digital resources

*“I look forward to such an organization of the literary records of medicine that a puzzled worker in any part of the civilized world shall in an hour be able to gain the knowledge pertaining to a subject of the experience of every other man in the world.”*

—George Gould, first president of the Association of Medical Librarians (now the Medical Library Association), May 1898

For over 100 years, the Medical Library Association (MLA) has upheld the belief that quality information is essential for improved health and has worked to ensure that health sciences librarians have the skills, knowledge, and leadership necessary for the delivery of information in a biomedical setting. The association has also promoted the concept of unrestricted, affordable, and permanent access of health information worldwide. For example, MLA's peer-reviewed *Journal of the Medical Library Association* *(JMLA)*, formerly the *Bulletin of the Medical Library Association (BMLA)*, has been made available since January 2000 on PubMed Central (PMC), a digital archive of the life sciences journal literature developed and managed by the National Center for Biotechnology Information (NCBI) and the United States National Library of Medicine (NLM). Access to PMC is free and unrestricted. Recently, NLM, working with MLA headquarters, made the full-text archives of *BMLA* from 1911 onward available online through PMC. This is an excellent resource for the study of health information sciences and the management of knowledge-based information, putting into practice MLA's belief in open access. The association has supported open access to information in several other ways, including memberships in the Scholarly Publishing and Academic Resources Coalition (SPARC) and the Information Access Alliance (IAA). MLA's statement on open access, found at http://www.mlanet.org/government/info_access/openaccess_statement.html, defines the association's position on this important topic.

However, open access increases the need for more sophisticated information management tools and systems, such as quality filtering and customization of clinical and research information at the point of need and decision-making. MLA is pursuing a number of initiatives that address the specific information needs of clinicians, healthcare students, biomedical researchers, and institutional leaders. Our members are excited to be in a unique position to develop tools, resources, and advice on how to find relevant information on the Internet. For example, MLA members have developed a *User's Guide to Finding and Evaluating Health Information on the Web* for the Pew Internet and American Life Project. The guide provides access and evaluation guidelines and MLA's top ten most useful Web sites, as well as lists of top Web sites for cancer, diabetes, and heart disease.

MLA is furthering the concept of evidence-based medicine through its exploration and definition of expert searching techniques (see http://www.mlanet.org/resources/expert_search/) and the provision of continuing education opportunities in this area (see http://www.mlanet.org/education/telecon/ebhc/index.html). These techniques identify best practices and cutting-edge clinical and research knowledge and cull through a sometimes overwhelming amount of medical literature that continues to grow exponentially. MLA's work in the area of expert searching was prompted by the increased emphasis on evidence-based practice by the Institute of Medicine. This, along with the publicity following the unfortunate death of a healthy research volunteer at Johns Hopkins about the need for more vigilance in maintaining the quality of literature searching, has created a renewed interest in the knowledge base and skill set required for expert literature searching and expert consultation. The use of evidence- or knowledge-based information retrieved through the expert searching process can help insure the clinical, administrative, educational, and research success and positive performance of the individual healthcare provider as well as the hospital or academic health center.

In addition to retrieving the best evidence, it is also important to deliver knowledge and services within the specialized context to patient care, research, and learning. MLA's exploration, along with NLM, of the informationist concept, i.e., specialist librarians who blend the knowledge and skills of both the clinical and information sciences, is defining new roles for librarians for providing filtered and customized clinical/research information at the point of need and decision-making (for more information, see http://www.mlanet.org/research/informationist/). Librarians are being recruited to join clinical and research teams as clinical medical librarians and information specialists in context and to provide expert consultation on issues ranging from informatics literacy to evidence-based medicine classes.

Besides health-care providers, millions of consumers search for health information on the Web every year. Recognizing the documented difficulties and frustrations health professionals and consumers face in coping with the barrage of available information in a way that results in informed healthcare decisions, MLA has established its health information literacy program (see http://www.mlanet.org/resources/healthlit/index.html) to stress the importance of “information” in health literacy. The association defines health information literacy as the set of abilities needed to recognize a health information need; to identify likely information sources and use them to retrieve relevant information; to assess the quality of the information and its applicability to a specific situation; and to analyze, understand, and use the information to make good health decisions. MLA has also developed a resources Web site for health consumers at http://www.mlanet.org/resources/consumr_index.html and http://caphis.mlanet.org/consumer/index.html that helps them find quality health information on the Web. These tools are publicly available to anyone in the world at any time.

MLA recognizes that this is a time of rapid change in our society in which the availability of digital resources in a networked environment provides unprecedented opportunities for more open access to the scientific and medical literature. As health sciences librarians, we are excited about the potential to serve a much wider group of international consumers, ranging from medical researchers to patients and their relatives. We will continue to work to convert access to information into access to knowledge.

## 

**Figure:**
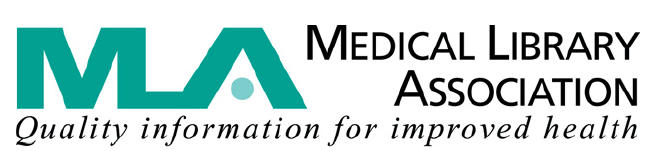
www.mlanet.org

## Abbreviations

*BMLA*: *Bulletin of the Medical Library Association*
IAA: Information Access Alliance
*JMLA*: *Journal of the Medical Library Association*
MLA: Medical Library Association
NCBI: National Center for Biotechnology Information
NLM: National Library of Medicine
PMC: PubMed Central
SPARC: Scholarly Publishing and Academic Resources Coalition
